# Review of ultrasound-guided labeling: exploring its potential in teaching cadaveric ligaments during anatomical dissection courses

**DOI:** 10.5116/ijme.65ae.4782

**Published:** 2024-01-31

**Authors:** Felix Margenfeld, Adib Zendehdel, Giorgio Tamborrini, Amélie Poilliot, Magdalena Müller-Gerbl

**Affiliations:** 1 Institute of Anatomy, University of Basel, Switzerland; 2 Swiss Ultrasound Center UZR and Institute for Rheumatology, Basel, Switzerland

**Keywords:** Medical education, scoping review, human cadaver, ultrasound, ligaments

## Abstract

**Objectives:**

This scoping review aimed to give an overview of the existing literature about ultrasound-guided labeling techniques of human cadaver ligaments and tried to work out the possibilities of integrating ultrasound into dissection courses.

**Methods:**

A literature review was carried out on the 3rd of January 2023, with relevant studies discovered in the following databases: MEDLINE, EMBASE, CENTRAL, BIOSIS Previews and Web of Science Core Collection. Grey literature was also considered. The reference lists of all relevant papers were scanned. Only ultrasound studies on human cadaver ligaments were included. The included studies' general characteristics and ultrasound-guided approaches to label the ligaments were taken from them and examined.

**Results:**

The search found 8899 matches, but only 96 of them met the criteria. The transverse carpal ligament (15.62%) and the annular pulleys (19.79%) were the ligaments that had received the greatest research attention. Twenty-three studies are included in the methodological analysis. Both the marking substrate and the injected volume were diverse. Although 65% of the included studies achieved 100% accuracy using the ultrasound directed labeling approaches.

**Conclusions:**

Ultrasound-guided labeling techniques achieve a high accuracy. Therefore, this methodology could be a potential teaching tool for students during the dissection course. But caution is advised in drawing general conclusions because of the small sample sizes and different methodologies in the studies. Future larger-scale research is necessary.

## Introduction

Skeletal ligament are a dense, fibrous bands of collagenous tissue that attache two bones to one another by expanding between them while remaining slightly stretchy.^1^ There are approximately 900 skeletal ligaments in the human body, and they vary in size, form, location and direction.

Microscopically, ligaments are mainly constructed of parallel collagen type I fibers. The assembly of polypeptide triple helices results in the formation of type I collagen fibrils, from which higher-order structures such as fibril bundles and fibers are constructed in the presence of both collagenous molecules and non-collagenous molecules, such as proteoglycans. The epiligament is a thin layer that surrounds the ligaments and is an essential source of cells and matrix metalloproteinases. It is crucial to the ligament's capacity to heal after injury.^2^ In particular, metalloprotein kinases 2 and 9 are of outstanding importance for scar tissue remodeling.^3^^, ^^4^

Ligamentous injuries are primarily traumatic, particularly during athletic activities, and the gold standard for detecting ligamentous rupture is MRI, or ultrasound in selected regions. Ultrasound (US) is suitable, in selected well-accessible regions, with equivalent sensitivity and specificity as MRI for rapid and reliable diagnosis of ligamentous pathologies.^5^^, ^^6^ As an inexpensive, portable diagnostic tool, it can also clarify dynamic issues in the musculoskeletal system and could be considered as the expansion of physical examination.^7^

Bedside examination is already being taught at the preclinical phase and is a significant, essential building component in medical studies. In the optimal instance, the student develops a physical examination that is capable of reliably ruling out possible differential diagnoses against the background of the increasing body of knowledge that they acquire in the course of their studies. Here, ultrasound makes it possible to expand the spectrum of differential diagnoses that can be ruled out. Consequently, it is important to promote the early use of this low-radiation and resource-saving diagnostic instrument, referred to as "the new stethoscope" in the medical field.^8^

There have been some new developments as technology has advanced.  Since equipment can now quickly connect to tablets through Wi-Fi or Bluetooth, integrating ultrasound into medical student education has never been simpler. Thereby, students profit from an early integration twice, expanding their anatomical knowledge and learning to handle ultrasound technology.^9^ The outcomes are spectacular; for instance, the anterolateral ligament of the knee may be seen using ultrasound on cadavers and can be precisely labeled using ultrasound guidance to guarantee preservation during dissection.^10^^-^^12^ A development that will probably persist is the implementation of ultrasound-based educational programs in medical schools.^13^ Especially, in the dissection course, which is still a pillar of the anatomical curriculum, clinical understanding and the teaching of clinical pathologies should be intensified.^14^ Radiologists recommend teaching radiology and anatomy in tandem during pre-clinical training to better prepare students for clinical practice.^15^ We believe that students might profit from the inclusion of ultrasonography as an essential, well-established, and versatile technique in the dissection course.

The aim of the current review study paper is to provide an outline of the existing ultrasonographic observations on ligaments of human cadavers. The three specific objectives of this scoping review were: to conduct a systematic search of the published and grey literature for ultrasonographic investigations on ligaments of human cadavers, map out the key features and ultrasound-guided labeling techniques of the identified articles, and identify novel directions that could progress the field of study.

## Methods

The methodology followed the PRISMA-ScR (Preferred Reporting Items for Systematic Reviews and Meta-Analyses extension for Scoping Reviews) guidelines.^16^ The review included the following five key phases: (1) identifying the research question, (2) identifying relevant studies, (3) study selection, (4) charting the data, and (5) collating, summarizing, and reporting the results.

### Research question

This review was guided by the question, “What are the general characteristics of the studies and the characteristics of US-guided labeling techniques on the present topic?” Therefore, a protocol was registered on November 28^th^, 2022, on OSF Registries.

### Search strategy

A comprehensive literature search was conducted by searching for relevant studies in the following databases: MEDLINE, EMBASE, CENTRAL, BIOSIS Previews and Web of Science Core Collection. Grey literature was also considered by two different ways: Regarding grey literature databases: National Grey Literature Collection. For PhD theses and dissertations, the databases EThOS and Open Access Theses and Dissertations were screened for relevant studies by combining the keywords used in the search strategy.

The reference lists of all relevant papers were scanned. Search process was performed on the 3^rd^ of January 2023. The search strategy was developed with the aid of and finally checked by a librarian.

### Citation management

Following the search, all identified citations were collated using Endnote,^17^ duplicates were removed by Endnote following Bramer, Giustini^18^ and manually with further duplicates removed when found later in the review process. Title and abstract screening were also performed in the Citation Manager Endnote.

### Eligibility criteria

Only ultrasound investigations in human cadavers were included, not animals or phantoms. The article was included, if the investigated subject was a ligament. For the analysis of the ultrasound approaches only B-Mode ultrasound was included. Sonoeleastography was not used because actively perfused "living" tissue and tissue under a certain tension would be necessary (e.g. tense or relaxed tendon). Furthermore, there are countless hardly comparable elastography techniques, which are not comparable with each other. At the present time, sonoelastography is not yet standardized and depends on the technique and the particular device. Doppler sonography is needed in the area of ligaments to measure pathological vascularization or to measure new vascularization, which makes little sense in cadavers. Since this work focuses on ligaments, intravascular, intraosseous, intraarticular ultrasound was deliberately omitted.

All types of relevant information including articles, PhD theses, dissertations and chapters in textbooks were considered. Only studies in English and German were included. The publication year wasn`t further restricted.

### Title and abstract screening

First title and abstract screening was performed for reviewing minimum inclusion criteria by one reviewer. References were added for full text screening if neither title nor abstract provide sufficient information. If uncertainties appeared, a second reviewer checked the references. Full text screening was performed by two reviewers. At each stage, disagreements were resolved by discussion or involvement of a third author. Anatomical structures were used to order the references. A criticall appraisal of the included sources of evidence and a risk of bias assessment of the included studies were not performed. But for publication bias, we checked whether the included studies registered a protocol.

### Data characterization

Data were extracted from the papers included in the scoping review by one reviewer manually. General characteristics extracted included author, year of publication, title, language, investigated ligament, journal and research question. Keywords were processed in the Endnote citation manager and if keywords were not transferred by creating a citation manager file, full texts were screened for missing keywords and keywords were completed. The characteristics of US-guided marking techniques that were extracted from the data were comprised of the marking substrate, injected volume, needle size, sample size, ultrasound expert credentials, dissection expert credentials and accuracy of the US-guided labeling technique. Measurements and ultrasound results of the included studies of the second part of the analysis were separately summarized.

### Data summary and synthesis of results

The data were further categorized in the Citation Manager Endnote. Then a spreadsheet was created and imported into Microsoft Excel 2019. A keyword analysis was performed by using VOS Viewer.^19^ Descriptive statistics were calculated to summarize the data. Frequencies and percentages were utilized to describe nominal data.

## Results

### Search and selection of included studies

**Figure 1:**
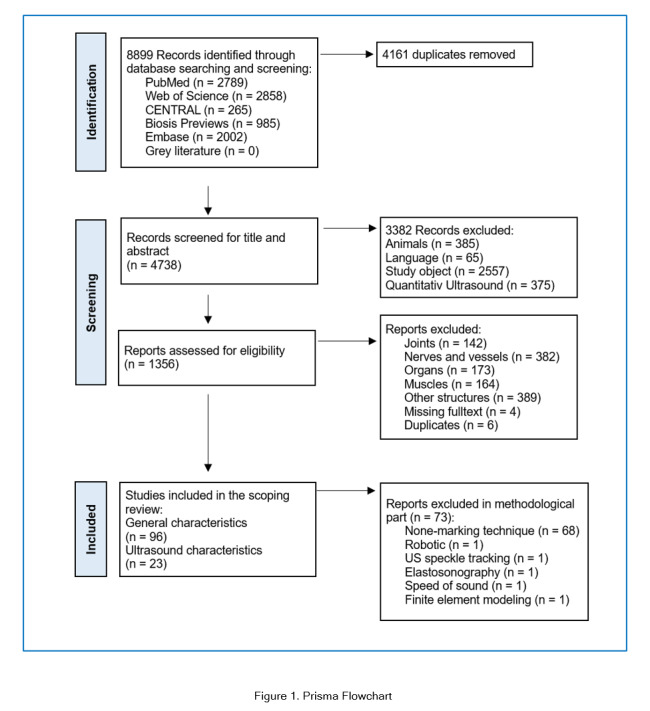
Prisma Flowchart

The search yielded 8899 results, of which 96 met the eligibility criteria. The authors agreed on all eligibility decisions on discussion without the need for a third party to be involved. Forward and backward citation tracking of the 96 included publications did not yield any additional publications. The PRISMA flowchart is presented in Figure 1.

### General characteristics of included studies

The general characteristics of the 96 studies are presented in Table 1. For keyword analysis VOS viewer was used. In 21 studies, keywords were missing and could not be included in analysis. The threshold for including a keyword in the analysis was set to 1. The largest network which could be generated with VOS viewer included 53 keywords. The main used keywords were ultrasound (n = 10), elbow (n = 4), lateral collateral ligament, carpal tunnel syndrome and ultrasonography (each of them n = 3).

More than an 80% of the included studies were published in 2010 or earlier. Leading journals were J Ultrasound Med (9.37%) and Skeletal Radiol (8.33%). All of the included studies were published in English. The most investigated ligament was the annular pulley with a fifth of the included studies (19.79%), followed by the transverse carpal ligament (15.62%) and ulnar collateral ligament (9.37%). A quarter of the included studies investigated US-guided labeling techniques (23.95%). Another quarter considered US as an imaging tool in general and their ability to present ligaments (23.95%). Here, the imaging characteristics on ultrasound of different ligaments were investigated, for example the sacroiliac ligaments^20^^,^^21^ or the scapholunate ligament.^22^ US-guided interventional procedures received the greatest research attention (32.29%), of which 11 dealt with release techniques of the transverse carpal ligament and another 11 dealt with release techniques at the annular pulley. Less examined ligaments were e.g., the intermetatarsal ligament^23^ or the anterior talofibular ligament.^24^

**Table 1:** General characteristics of included studies (n = 96)

Characteristic	n	%
Publication year		
< 2000	4	4.16
2000-2004	6	6.25
2005-2009	6	6.25
2010-2014	19	19.79
2015-2023	61	63.54
Journal (n ≥ 5)		
AJR Am J Roentgenol	5	5.20
J Ultrasound Med	9	9.37
PM&R	6	6.25
Skeletal Radiol	8	8.33
Other (n < 5)	68	70.83
Language		
English	96	100
German	0	0
Ligaments (n ≥ 4)		
Annular pulley	19	19.79
Anterolateral ligament	4	4.16
Coracohumeral ligament	4	4.16
Several ligaments	7	7.29
Transverse carpal ligament	15	15.62
Ulnar collateral ligament	9	9.37
Other (n < 4)	38	39.58
Research Question		
Diagnostic	16	16.66
Imaging tool in general	23	23.95
Interventional	31	32.29
US-guided labeling technique	23	23.95
Other	3	3.12

### US-guided labeling techniques

In the methodological analysis 23 studies were included. One third of the included studies used either latex or dye as a marking substance (30.43%, respectively 28.08%). The injected volume was specified in milliliters and varied between 0.1 mL and 3 mL. Some studies used “drops” as an alternative unit (ca. 13%). The most commonly used needle size was between 21- and 25-gauge in around half of the included studies (47.82%). One fifth of the included studies did not specify anything (21.73%), and another fifth used needles smaller than or equal to 20-gauge (21.73%). Nearly 70% of the included studies used sample sizes between 1 and 10.

In more than half of the included studies radiologists performed the ultrasonograms (56.52%). Only in around 20%, the qualification was not described. In one study, a physical medicine and rehabilitation specialist scanned the ligaments.

In 25% of the instances, a surgeon and in 25% of the cases, an anatomist, performed the dissection. The qualifications of the dissector were not mentioned by the rest. The accuracy of the US-guided labeling techniques was high. 65.21% of the included studies achieved a value of 100%. Only one study did not mention the accuracy. The minimal precision that

could be reached was 60%, while about 17% of the listed experiments attained values below 80%. In Table 2, the characteristics of US-guided labeling techniques of the included studies are summarized.

**Table 2:** Characteristics of US-guided marking techniques of the included studies (n = 23)

Characteristic	n	%
Marking substrate		
Dye	6	28.08
Ink	2	8.69
Latex	7	30.43
Needles	3	13.04
Other	5	21.73
Injected volume		
1-2 drops	2	8.69
3-4 drops	1	4.34
0.1 mL	4	17.39
0.5 mL	3	13.04
1 mL	1	4.34
1.5 mL	1	4.34
2 mL	1	4.34
3 mL	1	4.34
n/c	9	39.13
Needle Size		
≤ 20-gauge	5	21.73
21-25-gauge	11	47.82
≥ 26-gauge	2	8.69
n/c	5	21.73
Qualification of Ultrasonographer		
Investigator (qualification not described)	2	8.69
Investigator (with experience)	4	17.39
Radiologist	13	56.52
Physical medicine and rehabilitation specialist	1	4.34
n/c	3	13.04
Sample Size		
1-5	11	47.82
6-10	6	26.08
11-15	2	8.69
≥ 16	4	17.39
Dissector		
Anatomist	6	26.08
Surgeon	6	26.08
n/c	11	47.82
Accuracy		
60-70%	1	4.34
71-80%	3	13.04
81-90%	2	8.69
91-99%	1	4.34
100%	15	65.21
n/c	1	4.34

## Discussion

The first scoping review to address the topic of ultrasonography examinations of ligaments from body donors is presented in this study.  It shows a wide field of clinical and anatomical applications. To examine novel injection techniques on peripheral nerves and the spread behavior of the injected substrate, US-based labeling approaches on human cadavers have been well-established in the literature, particularly in the anesthesiology field.^25^^-^^27^ But as our scoping analysis shows, ultrasound-guided labeling is quite accurate even for ligaments.

The marking substrate had no bearing and there was no discernible relationship between needle size and volume. The labeling technique is an effective tool to safeguard ligaments that are hard to recognize throughout the dissection course, such as the anterolateral ligament, which was one of the most US-guided labeled ligaments. It was successfully marked in four of the included studies of this scoping review. Considering that there is still an inconsistent opinion about the anterolateral ligament regarding prevalence, femoral origin, tibial insertion and relation to the surrounding structures,^28^^, ^^29^ further studies with ultrasound-guided labeling techniques could be used to help clarify the situation. Investigating such questions using US-guided labeling approaches is interesting because its application in the dissection course is made easier and more resource-efficient. Indeed, even small quantities (0.1 mL or a few drops) are adequate to correctly designate a ligament.^30^^, ^^31^ A further advantage is that needles can be reused or multiple ligaments from different body donors can be marked with the same needle, since disinfection plays only a minor role in body donations. Taking this idea even further, different elusive anatomical structures could be marked in different colors, which might assist the student to comprehend them more effectively and memorize the structure.^32^

A precise diagnosis, musculoskeletal ultrasonography, in-depth anatomical knowledge and, in particular, significant practical injection abilities are necessary for the right injection method. In the case of pre-interventional precise identification of the injection site or for executing an injection under direct supervision utilizing various modalities, high-resolution musculoskeletal ultrasonography is essential. Encouraging ultrasound-guided anatomically-precise labeling techniques in student dissection courses seem to increase the understanding of ligamentous function in clinical practice. Ideally, in the context of dissection and labeling, the corresponding ligament can also be functionally tested and visualized via ultrasound in the same setting.^33^^, ^^34^

For example, the popliteofibular ligament - an essential stabilizer of the posterolateral corner of the knee - was studied in two searches.^35^^, ^^36^ The study of Pekala, Mizia^35^ revealed that there was no statistically significant difference between ultrasonography and cadaveric dissection for the average ligament length or width at the fibular head insertion and at the junction of the popliteus muscle. An ultrasound examination of a cadaver allowed for the effective visualization of the arcuate sign caused by a fibular styloid process fracture. The study of Sekiya, Jacobson^36^ assessed seven cadaveric knees via ultrasound with a 10- and 12-MHz linear transducer and were also able to identify all structures of the posterolateral corner of the knee, including the posterofibular ligament. This delicate but crucial ligament is a good candidate for labeling testing in subsequent studies, as it is sometimes difficult to see during the dissection phase.

However, ultrasound guidance has great potential not only for difficult-to-dissect structures such as the posterofibular ligament. It is also of interest for testing or improving various surgical procedures. The annular ligaments of the hand have attracted a large research interest.  This is probably due to the fact that trigger finger remains one of the most common causes of consultations for hand pain and is particularly evident in diabetics with an HbA1c greater than 7% with a prevalence estimated to range between 5% and 20%.^37^ Our analysis reveals that in a pilot cadaveric study, principally ultrasound-assisted percutaneous trigger finger release procedures were evaluated for practicality and dependability on cadaveric fingers. As demonstrated by the work of Rajeswaran, Lee^38^ these may be effectively applied in the clinic and are equivalent to open surgical care.^39^ In addition, as carpal tunnel syndrome is the most prevalent entrapment neuropathy, the transverse carpal ligament has attracted a lot of study interest.^40^ Here, the US-assisted release technique could be tested on cadavers and represents a reliable alternative treatment for carpal tunnel syndrome.^41^

A further interesting study by Delforge, Lecoq^42^ indicates that ultrasound practice on cadavers is not only beneficial to trainees, but also to surgical technique training.  They dissected 20 coracoacromial ligaments under ultrasonographic guidance, sparing the acromial branch of the thoracoacromial artery. The average US-guided surgery lasted 18.5 minutes, with 17 sections completed (85%) and 3 parts left unfinished (15%). Only the anterolateral bundle of the coracoacromial ligament could be seen in each of the three incomplete sections; the posteromedial bundle was not visible. In 90% of the instances (n = 18), the acromial branch of the thoracoacromial artery could be preserved.

Identifying new avenues of research with the potential to advance the field was another aim of our scoping review. Here for instance, the coracoclavicular ligament, as with many other ligaments, has not yet been sonographically investigated on cadavers despite its clinical significance in the Rockwood classification of acromioclavicular joint injuries. In contrast, the coracohumeral ligament has been studied several times, including its identification via ultrasound,^43^ injection approaches^31^^,^^44^ and US-guided percutaneous sectioning technique.^45^

### Strengths and limitations

In the current text, all processes were conducted with rigor and transparency. It followed a protocol that was listed in the OSF Registries. To provide a complete search of the literature, five digital bibliographical databases and a grey literature search were incorporated in the course of the investigation. Since the initial goal was to examine all ultrasound tests conducted on body donations, the search terms were purposefully broad. We elected to focus on assessing a portion of the data since there were plenty of data. As a result, there were many studies found by the search, which made the title and abstract screening process time-consuming and perhaps mistake-prone. The most common terms for ultrasound (ultrasonography and ultrasound) were included in our search strategy, according to a key analysis. The term cadaver was underrepresented (n = 2). However, seven phrases previously used to describe a cadaver were included in our search methodology; but other terms, such as specimen, also surfaced and were not included in our search request. Scoping reviews do not adhere to the same strict standards that systematic reviews do, and there is no risk of bias assessment, which leaves space for biases like selection bias. Publication bias is strong since none of the studies included in the methodological analysis registered a visible protocol prior to investigation. Generally, caution should be taken when drawing conclusions from scoping reviews because they frequently summarize the findings without fully synthesizing the results.

Another content limitation of our analysis is that we included also the retinacula, but Stecco, Macchi^46^ showed in their histological analysis of the flexor retinaculum and the transverse carpal ligament at the wrist that a retinaculum should be considered more as a reinforcement of the deep fascia and has a totally different histological structure compared to a skeletal ligament, which main function is to connect bones with each other.

## Conclusions

The present study evaluates a range of ligaments and we believe it is the first comprehensive review to screen ultrasound studies of human cadaveric ligaments. Although the ultrasound-guided labeling procedures covered a wide range of subjects, they all produced ligament labels with a high degree of accuracy. But, caution is advised in drawing general conclusions because of the small sample sizes and different methodologies in the studies. We support the early use of ultrasound as a teaching tool for anatomy but further comparative and larger-scale studies should be conducted to evaluate and strengthen the benefits to students. The current review showed that US guided labeling techniques can help students to grasp and retain small structures such as ligaments during the dissection course and therefore the recommendation of an early implementation of ultrasound in learning anatomy is made.

### Conflict of Interest

The authors declare that they have no conflict of interest.
